# Impact of an AI-based laparoscopic cholecystectomy coaching program on the surgical performance: a randomized controlled trial

**DOI:** 10.1097/JS9.0000000000001798

**Published:** 2024-06-20

**Authors:** Shangdi Wu, Ming Tang, Jie Liu, Dian Qin, Yuxian Wang, Siwei Zhai, Enxu Bi, Yichuan Li, Chunrong Wang, Yong Xiong, Guangkuo Li, Fengwei Gao, Yunqiang Cai, Pan Gao, Zhong Wu, He Cai, Jian Liu, Yonghua Chen, Chihua Fang, Li Yao, Jingwen Jiang, Bing Peng, Hong Wu, Ang Li, Xin Wang

**Affiliations:** a Department of General Surgery, Division of Pancreatic Surgery, West China Hospital of Sichuan University; b West China School of Medicine, West China Hospital of Sichuan University; c ChengDu Withai Innovations Technology Company, Chengdu; d Department of General Surgery, Qingdao West Coast New Area Central Hospital, Qingdao, Shandong; e Hepatobiliary and Pancreatic Surgery, Guang’an People’s Hospital, Guang’an; f Department of Hepatobiliary Surgery, Xuanhan People’s Hospital, Dazhou; g Hepatobiliary Department, Panzhihua Central Hospital, Panzhihua; h Department of Hepatobiliary Surgery, Chengdu Second People’s Hospital; i Department of General Surgery, Liver Transplant Center, West China Hospital, Sichuan University; j Department of General Surgery, Chengdu Fifth People’s Hospital, Chengdu, Sichuan; k Department of Hepatobiliary Surgery, Zhujiang Hospital, Southern Medical University, Guangzhou; l Department of Colorectal Surgery, China-Japan Friendship Hospital, Beijing; m Med-X Center for Informatics, Sichuan University, Chengdu, People’s Republic of China

**Keywords:** artificial intelligence, cholecystectomy, critical view of safety, surgical coaching

## Abstract

**Background:**

Laparoscopic cholecystectomy (LC) is the gold standard for treating symptomatic gallstones but carries inherent risks like bile duct injury. While the critical view of safety (CVS) is advocated to mitigate bile duct injury, its real-world adoption is limited. Additionally, significant variations in surgeon performance impede procedural standardization, highlighting the need for a feasible, innovative, and effective training approach. The aim of this study is to develop an artificial intelligence (AI)-assisted coaching program for LC to enhance surgical education and improve surgeon’s performance.

**Materials and methods:**

The authors conducted a multicenter, randomized controlled trial from May 2022 to August 2023 to assess the impact of an AI-based coaching program, surgical coaching program, on novice performing LC. Surgeons and patients meeting specific inclusion criteria were randomly assigned to either a coaching group with AI-enhanced feedback or a self-learning group. The primary outcome was assessed using the Laparoscopic Cholecystectomy Rating Form, with secondary outcomes including surgical safety, efficiency, and adverse events. Statistical analyses were performed using SPSS, with significance set at a *P*-value less than 0.05.

**Results:**

Between May 2022 and August 2023, 22 surgeons were initially enrolled from 10 hospitals, with 18 completing the study. No demographic differences were noted between coaching and self-learning groups. In terms of surgical performance (Laparoscopic Cholecystectomy Rating Form scores), the coaching group showed significant improvement over time (31 to 40, *P*=0.008), outperforming the self-learning group by study end (40 vs 38, *P*=0.032). Significant improvements in CVS achievement were also noted in the coaching group (11% to 78%, *P*=0.021). Overall, the coaching program was well-received, outpacing traditional educational methods in both understanding and execution of CVS and participants in the intervention group expressed strong satisfaction with the program.

**Conclusions:**

The AI-assisted surgical coaching program effectively improved surgical performance and safety for novice surgeons in LC procedures. The model holds significant promise for advancing surgical education.

## Introduction

Highlights

- An artificial intelligence-based surgical coaching program (SmartCoach) for laparoscopic cholecystectomy training was established.
- We conducted a prospective multicenter randomized controlled trial research to explore the efficacy of SmartCoach.
- SmartCoach system enhanced surgeons’ performance and surgical safety compared to traditional self-learning methods.
- An artificial intelligence-based coaching models showed potential in surgical education.

Laparoscopic cholecystectomy (LC) stands as the gold standard for treating patients with symptomatic gallstones and is one of the most frequently performed procedures in general surgery. Annually, it accounts for ~750 000 cases in the United States alone^1^. Despite its standardization and over three decades of refinement, LC carries inherent risks, including bile duct injury (BDI)—a grave complication with a reported incidence of 0.3–1.5%^2,3^. This complication not only results in potentially irreversible harm to patients but also places substantial burdens on healthcare providers and systems. To mitigate the risk of BDI, achieving a critical view of safety (CVS) is recommended by numerous international guidelines^3,4^. Existing studies have shown that achieving CVS is technically feasible in ~90% of cases and has the potential to reduce the incidence of BDI to as low as 2 per 1 000 000 surgeries^5–7^. However, real-world clinical application often falls short, as evidenced by a prospective study in Strasbourg, which found a CVS attainment rate of merely 15.9%^8^. Our previous analysis using validated machine learning algorithms on multicenter datasets uncovered an even lower CVS achievement rate of 4.33%, with significant variations among surgeons^9^. This suggests that despite the relative procedural simplicity of LC, there exists a significant gap in systematic educational training.

Traditional surgical training paradigms continue to face challenges, particularly in connecting theoretical knowledge with practical application and providing timely, personalized feedback in clinical environments^10^. These gaps can jeopardize surgical safety and consistency, underscoring the urgent need for innovative, evidence-based educational models to ensure high-quality surgical outcomes and patient safety. To facilitate the transition from medical student to physician, new personalized education models have been developed, such as customized coaching programs^11^. A meta-analysis has shown the effectiveness of this approach, concluding that video-based surgical coaching can significantly improve technical performance^12^. However, the extensive time required for video review and data analysis can extend the learning curve for novice surgeons.

The advent of technologies such as artificial intelligence (AI) and deep learning has propelled the development and preliminary adoption of digital surgery^13^. Various studies have developed reliable models capable of automatically identifying surgical phases, anatomical structures, and instruments with high accuracy, demonstrating strong potential for implementation^14–16^. Our prior research has demonstrated the clinical utility of an intelligent system for automated analysis of LC videos, providing multidimensional data including surgical phases, disease severity, critical actions, and CVS scores^9^.

Building on this system and leveraging existing surgical coaching methodologies, we conducted a prospective randomized controlled trial (RCT) to evaluate the efficacy of an AI-based coaching system for LC in enhancing surgical safety and quality for novice surgeons.

## Methods

### Study design and participants

A multicenter, paralleled, open-label RCT was performed to assess the impact of an AI-based coaching program named surgical coaching program (SmartCoach) on overall performance and safety in surgery among novice surgeons from May 2022 to August 2023. This study was designed according to the Consolidated Standards of Reporting Trials–Artificial Intelligence extension^17^. The consent of surgeons and patients was acquired. The research was approved by the Ethics Committee on Biomedical Research and was registered with the Chinese Clinical Trial Registry.

To better evaluate the impact of the SmartCoach program on surgeons in the learning curve, videos of participants were assessed by hepatobiliary experts. Those who have been deemed to meet the SmartHelp level according to the Zwisch scale were included in this study^18^. Surgeons at the SmartHelp level are capable of alternating between the roles of lead surgeon and first assistant, and they have demonstrated the ability to execute key components of the operation under the supervision of attending surgeons. Meanwhile, patients must meet specific requirements, including 1) an age of 18 years or older, 2) a confirmed surgical indication for LC, and 3) no other concomitant procedures during this hospital stay. Patients with severe comorbidities, including cardiopulmonary insufficiency, high risk of bleeding, and suspect of gallbladder malignancy or acute gallbladder inflammation (Parkland score ≥3), were excluded^19^. Besides, in order to more objectively and accurately assess the surgery, videos must meet the following criteria: 1) videos should contain complete surgical phases and without conversion to open surgery or taken over by a senior surgeon; 2) videos must be standard laparoscopic recordings featuring a tubular zoomed view, a minimum resolution of 720×560 with a frame rate of 25 frames per second. Videos containing other nonstandard procedures/techniques, such as fluorescence, were excluded. A senior attending surgeon who received coaching training and has extensive experience in LC was recruited as a coach in this study.

### Randomization and masking

For randomization, eligible surgeons with SmartHelp level were assigned by a web-sited randomization system that used block randomization method. Participants were assigned to either Coaching group or Self-learning group. The blind method did not apply in the current study due to the characteristics of the intervention.

### AI-based coaching system-SurgSmart

Our team used an AI algorithm known as SurgSmart, which accurately identifies surgical phases, disease severity, critical duct division action, and CVS score^9^. Through two iterative improvements, the overall accuracy rate has reached an impressive 90%, with results being presented in the form of comprehensive surgical reports (Supplementary Fig. S1 in Appendix 1, Supplemental Digital Content 1, http://links.lww.com/JS9/C817). Simultaneously, SurgSmart has undergone an update to enhance its intelligent visualization capabilities, enabling in-depth analysis and review of pivotal events. This is achieved by automatically generating synchronized key timelines beneath surgical videos (Supplementary Fig. S2 in Appendix 1, Supplemental Digital Content 1, http://links.lww.com/JS9/C817), simplifying the educational process in the SmartCoach study.

To effectively deploy the AI algorithms, an adequate computing system is required. The CPU should be a multi-core CPU (Intel Core i5-13600KF), 16GB+ DDR4/DDR5 RAM for memory-intensive tasks, a 500 GB+NVMe SSD for speedy data access, and a high-end GPU (A5000) for accelerated neural network computations. These components together ensure optimal performance for immense matrix math operations inherent in neural networks.

### Coaching workflow

**Figure 1:**
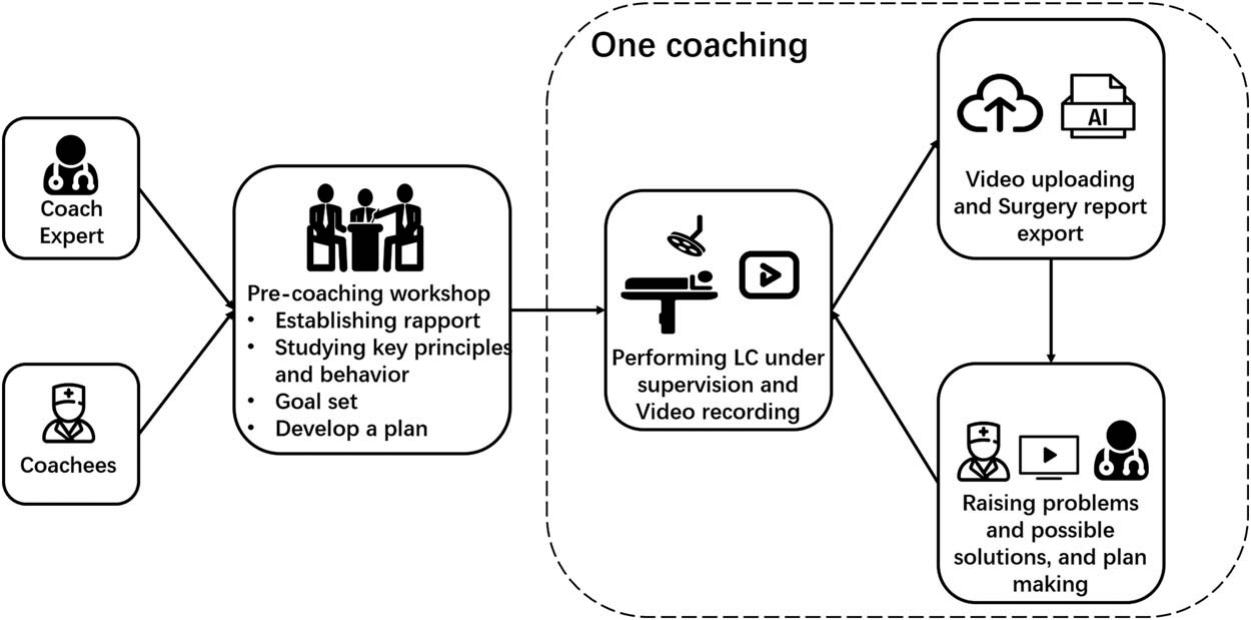
SmartCoach workflow. SmartCoach, surgical coaching program.

Based on previous coaching principles and models^20^, we constructed the SmartCoach program workflow (Fig. 1). In detail, participants were randomized to a coaching group (intervention) or self-learning group (control). Each coachee performs five LC procedures under supervision as part of the study. After each surgery, the participants will engage in a 30 min online coaching session or self-learning activity, depending on the groups. For participants in the coaching group, the coach and coachees were all required to learn the core principles and activities of surgical coaching before coaching sessions. Surgical reports will be immediately generated after every surgery, and a coaching session will ensue (Supplementary Fig. S1 in Appendix 1, Supplemental Digital Content 1, http://links.lww.com/JS9/C817). This session involved in-depth discussions between the coach and coachees: efficient debriefing is conducted based on the surgical report and intelligent visualization system to pinpoint problems, which are then provided as feedback to the coachees while formulating subsequent improvement plans for the next surgery. For participants in the self-learning group, after each surgery, coachees will have access to online surgical datasets (without surgical reports) or textbooks to learn independently. To better understand the coachees’ comprehension of the training system and their satisfaction with this study, a 5-point Likert scale (from Not at all to Extremely) questionnaire was distributed before and after program (Appendix 2, Supplemental Digital Content 2, http://links.lww.com/JS9/C818).

### Study outcomes

The primary outcome was the Laparoscopic Cholecystectomy Rating Form (LCRF)^21^, a comprehensive, procedure-specific operative performance rate system, which is assessed by two well-trained, experienced hepatobiliary surgeons. Disagreements were solved by consulting a third expert. Secondary outcomes included metrics for assessing surgical safety (CVS score), efficiency (surgical phase duration, phase transition times, and overall surgical duration), and intraoperative adverse events (such as bleeding, tissue injury, and so on)^22^. Additionally, surgeons’ demographic data and patients’ perioperative data were collected and analyzed.

### Statistical analysis

For the primary outcome of LCRF, we anticipated a 5-point improvement with a standard difference of 3.0^21^. Our goal was to recruit at least 22 surgeons (11 for each study arm), considering that 90% power at a 0.05 significance level to detect a difference in LCRF scoring. Power calculation was done using Power Analysis and Sample Size Software (NCSS). We report continuous variables as the mean±SD and others as the median (25–75 quantiles). All normally distributed variables were expressed as the mean±SD while the non-normally distributed variables were the median (25–75 quantiles). All statistical results were considered statistically significant at a *P*-value less than 0.05 (both sides). Wilcoxon signed-rank test was used to compare LC1 and LC5 within the same group. Mann–Whitney *U* test was used to detect the difference between groups. SPSS Statistics, v26.0 (IBM Corp.), was used to analyze data.

## Results

### Study participants

**Figure 2:**
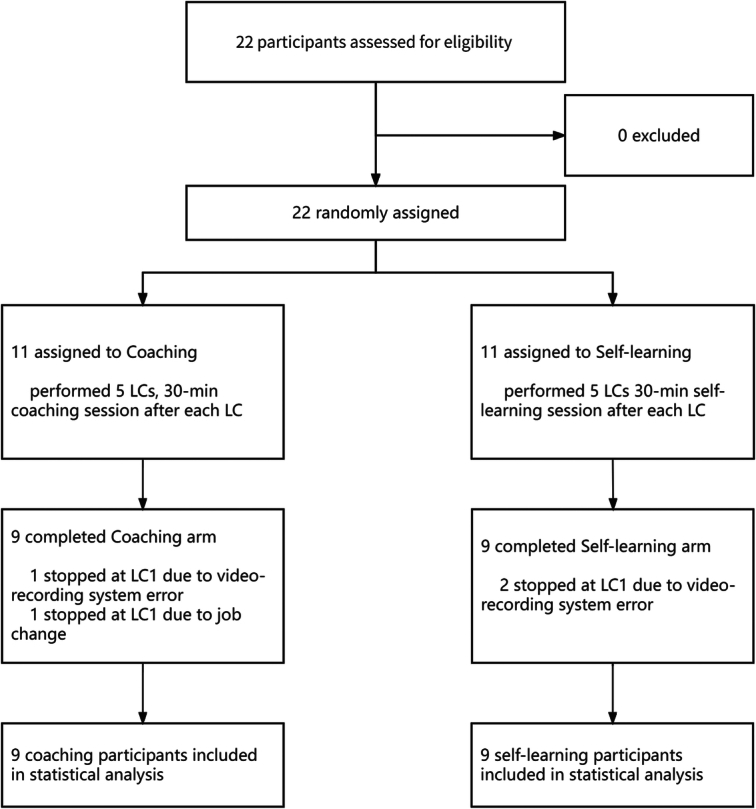
CONSORT flowsheet. LC, laparoscopic cholecystectomy.

From May 2022 to August 2023, 22 participants from 10 hospitals were randomly assigned to the coaching group or self-learning group. In the coaching group, one surgeon withdrew from the study due to job-related changes, while another exited the study due to issues with video recording equipment. In the self-learning group, two surgeons withdrew from the research due to video recording equipment problems. Ultimately, 18 surgeons participated in this study, with nine surgeons in each of the coaching and self-learning groups, collectively completing a total of 90 surgeries throughout this research (Fig. 2 study flow chart).

Demographic characteristics of the participants are presented in Table 1. No significant differences were observed between the two arms. Specifically, almost all participating surgeons were male, except for one female surgeon in the self-learning group. In both groups, residents constituted the majority of participants, accounting for 78% of the corresponding group. Interestingly, all participants (100%) in the self-learning group were from teaching hospitals. In contrast, only five participants (56%) in the coaching group were from teaching affiliated hospitals, which further leads to the surgeons in the self-learning group having more opportunities for surgical participation compared to those in the coaching group. Additionally, the median practicing duration for surgeons in the coaching group was 2 years, while for those in the self-learning group, the median practicing duration was 3 years. Lastly, to ensure comparable baseline skills, both groups included doctors at the SmartHelp level.

**Table 1:** Demographic characteristics of participants.

	Coaching (*N*=9)	Self-learning (*N*=9)
Sex at birth		
Male	9 (100%)	8 (88.9%)
Female	0 (0%)	1 (11.1%)
Age	32 (28–33)	32 (28–37)
Title of surgeons		
Resident	7 (78%)	7 (78%)
Fellow	2 (22%)	2 (22%)
Hospital characteristics		
Nonteaching affiliated hospital	4 (44%)	0 (0%)
Teaching affiliated hospital	5 (56%)	9 (100%)
Practicing years	3.0 (2.0–4.0)	2.0 (1.0–4.0)
Skill level^a^	SmartHelp	SmartHelp

a Skill level was assessed by the Zwisch scale.

### Coaching outcomes

**Figure 3:**
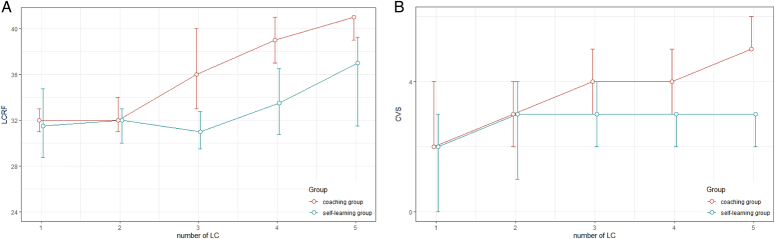
A, B, Changes of LCRF and CVS score from baseline to the last LC for both arms. The red line is the coaching group; the green line is the self-learning group. CVS, critical view of safety; LCRF, laparoscopic cholecystectomy rating form.

All intraoperative-related outcomes in this study are detailed in Table 2 and Supplementary Table S1 in the Appendix 1 (Supplemental Digital Content 1, http://links.lww.com/JS9/C817). For the primary outcome in this study, specifically, the LCRF reflecting the overall surgical performance, no significant differences were observed between the coaching group and the self-learning group at the initial stage (median, 31 vs 34, *P*=0.249). In the coaching group, participating surgeons showed a significant improvement in their last LC surgery compared to their first (median, 31 vs 40, *P*=0.008). However, no significant difference was observed in the self-learning group (median, 34 vs 38, *P*=0.138). Additionally, after completing the learning phase, the overall score for the coaching group was significantly higher than that for the self-learning group (median, 40 vs 38, *P*=0.032) (Fig.3A).

**Table 2:** Changes from baseline in surgical performance between coaching and self-learning participants.

	LC-1		LC-5		*P* ^a^		*P* ^b^	
Variable	Intervention	Control	Intervention	Control	Intervention	Control	LC1	LC5
LCRF	31 (29–32)	34 (31–37)	40 (39–41)	38 (32–39)	0.008	0.138	0.249	0.032
CVS score					0.021	1.000	0.303	0.004
＜5	8 (89%)	9 (100%)	2 (22%)	8 (89%)				
≥5	1 (11%)	0 (0%)	7 (78%)	1 (11%)				
Surgical phase length								
IT	411 (355–591)	400 (178–781)	700 (492–787)	496 (150–876)	0.015	0.767	0.757	0.453
EA	85 (63–122)	101 (66–108)	82 (63–129)	100 (80–108）	0.594	0.859	1.000	0.965
AL	0 (0–235)	26 (0–263)	23 (0–54)	0 (0–168）	0.400	0.499	0.604	0.962
MHT	745 (647–1037)	990 (753–1123)	546 (438–656)	492 ( 430–684)	0.038	0.066	0.310	0.965
DGB	337 (215–401)	163 (151–300)	188 (66–474)	134 (101–322)	0.441	0.515	0.270	0.507
EG	54 (30–98)	0 (0–58)	100 (78–140)	86 (0–243)	0.515	0.463	0.387	0.657
COR	253 (152–532)	362 (173–538)	287 (60–376)	275 (142–362)	0.678	0.953	0.791	0.566
Times of surgical phase switch	19 (14–20)	15 (10–21)	10 (10–16)	10 (10–14)	0.068	0.047	0.691	0.893
Effective operative time	1998 (1491–2510)	1587 (1390–2260)	1251 (890–1902)	1320 (891–1761)	0.066	0.260	0.566	0.965
iAE^c^					0.854	0.144	0.053	0.963
Total	16	8	15	16				
Bleeding	15 (94%)	8 (100%)	13 (87%)	13 (81%)				
Injury	1 (6%)	0	2 (13%)	3 (19%)				
Cautery	0	0	0	0				
Arrhythmia	0	0	0	0				

a LC1 vs LC5 in coaching group or self-learning group.

b Comparison of LC1 or LC5 between two groups.

c iAE was calculated according to ClassIntra criteria.

AL, adhesion lysis; COR, clear the operative region; CVS, critical view of safety; DGB, dissecting gallbladder from liver bed; EA, establishing access; EG, extracting the gallbladder; iAE, intraoperative adverse event; IT, idle time; LCRF, laparoscopic cholecystectomy rating form; MHT, mobilizing the hepatocystic triangle.

In terms of surgical safety, at the initial stage of the study, most participating surgeons (89 vs 100%, *P*=0.303) in both the coaching and self-learning groups were unable to achieve the desired CVS score (≥5 points). After having undergone the coaching program, the CVS completion rate among surgeons in the coaching group had shown a significant improvement (from 11 to 78%, *P*=0.021), whereas no significant change had been observed in the CVS completion rate among those in the self-learning group (from 0 to 11%, *P*=1.000). Likewise, at the end of this study, the CVS completion rate in the coaching group was significantly higher than in the self-learning group (78 vs 11%, *P*=0.004) (Supplementary Table S3, Supplemental Digital Content 1, http://links.lww.com/JS9/C817 Fig. 3B and Supplementary Fig. S3 in the Appendix 1, Supplemental Digital Content 1, http://links.lww.com/JS9/C817).

As shown in Table 2, in terms of overall surgical efficiency, there was no significant difference between the two groups. However, when examining specific surgical phases, surgeons in the coaching group reduced the time spent on mobilizing the hepatobiliary triangle (745 to 546, *P*=0.038), and increased the idle time (411 to 700, *P*=0.015). Additionally, there were no significant differences between the two groups in terms of the number of phase transitions and effective surgical time. Lastly, regarding intraoperative adverse events (iAE), no significant differences were observed between the two groups.

In the questionnaire survey shown in Supplementary Table S2 in the Appendix 1 (Supplemental Digital Content 1, http://links.lww.com/JS9/C817) we found that both the coaching group surgeons and the self-learning group surgeons had previously lacked any form of surgical coaching. (Q11) Compared to the self-learning group surgeons, the coaching group surgeons demonstrated a significant improvement in both their understanding of CVS (from 2.78 to 4.22, *P*=0.028) and compliance with CVS execution (from 2.56 to 4.78, *P*=0.011) during the course of this study. After completing all surgical coaching activities, the coaching group surgeons uniformly expressed that the surgical coaching program was significantly superior to traditional surgical education methods such as lectures (4.33), video reviewing (4.11), and curricula (3.78). Furthermore, all coaching group surgeons expressed a high level of satisfaction with the SmartCoach, with ratings close to the maximum score (4.89).

## Discussion

Surgical quality reflects a surgeon’s understanding of surgery, technical proficiency, and intraoperative decision-making ability. It is closely related to patient outcomes^23,24^. Early training of young surgeons has a significant impact on their learning curve, intraoperative decision-making, and the standardization of operating procedures. Therefore, a scientifically validated training model is ultimately crucial for ensuring surgical quality and improving patient outcomes^10^. Several studies have demonstrated that surgical coaching outperforms traditional training methods, such as courses and simulators, enabling surgeons to reach higher levels of performance more efficiently^25,26^. However, the development of high-quality surgical coaching programs requires considerable expertise and time investment, which restricts their widespread availability. Currently, only a few centers in developed Western countries offer such programs^26–28^. In this context, our previous study explores the potential of the SurgSmart system, an AI-based platform designed to facilitate the ‘visualization’ and ‘digitization’ of surgical processes^9^. This system enhances the capability of surgical coaches to deliver effective training. In this study, it was observed that the surgeons who received AI-assisted training improved their overall surgical performance safety, showing the feasibility, efficiency, and effectiveness of the AI-based surgical coaching model in improving the performance of young surgeons.

For an effective SmartCoach, establishing an objective, quantifiable standard that comprehensively reflects surgical quality is crucial. This allows for individualized assessments of surgeons and provides immediate feedback, ultimately achieving the goal of surgical coaching^20^. Current assessment criteria in surgical coaching include the Objective Structured Assessment of Technical Skills (OSATS)^29^, the Global Operative Assessment of Laparoscopic Skills (GOALS)^30^, and so on. However, these criteria still incorporate a significant degree of subjectivity and can be difficult to quantify consistently. Despite systematic training efforts, ensuring that coaches consistently deliver objective and accurate evaluations of surgeons’ performance remains a challenge. After completing the program, a 2-point median difference in LCRF score between the two groups seemed not clinically different. Yet the CVS results showed a significant difference in achievement between coaching and self-learning participants (78 vs 11%, *P*=0.004), which is critical to ensure patients’ safety. We assumed that as a 10-item scale, the LCRF total score hid the safety problem caused by cognition deficiency of CVS to some extent, rather than surgical technique.

In this study, we emphasize surgical safety and process as key indicators of surgical quality. To quantify and assess these aspects in LC, we selected two metrics: CVS scores and surgical phases. The CVS scores, integral to ensuring procedural safety, are derived from established research methodologies. These scores assess three dimensions of CVS, each rated between 0 and 2. A score of 5 or higher indicates successful completion of CVS, meeting safety standards^9^. The surgical phases, on the other hand, serve as a direct manifestation of the surgical workflow. The duration of each phase, the sequence, and the frequency of phase transitions quantitatively reflect the flow of operation and knowledge of the procedure. In a standardized surgical report (Supplementary Fig. S1 in the Appendix, Supplemental Digital Content 1, http://links.lww.com/JS9/C817), we can instantly view the quantified CVS scores and surgical phase information postoperatively. Therefore, based on the detailed quantitative metrics mentioned above, surgical coaches can efficiently engage in coaching activities with coachees. They can provide personalized feedback on surgical safety and efficiency-related information, helping coachees set better goals and navigate the learning process more efficiently.

With the continuous advancement of AI technologies, particularly in areas such as computer vision and deep neural networks, numerous research institutions have begun exploring their potential applications within the realms of surgical quality control and education^31,32^. For AI technology to ultimately find practical applications in surgical education, it must meet two essential prerequisites. Firstly, there must be sufficiently accurate AI algorithms capable of digitizing surgery. This includes achievements by Harvard and Japanese teams in achieving recognition accuracy over 90% in surgical phases^14^ and one study achieving an 80% accuracy rate in CVS recognition^33^, demonstrating the feasibility of algorithms in recognizing complex surgical contents. Building upon the initial development of SurgSmart^9^, our team has continuously iterated the algorithms by incorporating high-quality data. As a result, we have achieved an average accuracy rate of over 90% in algorithms for LC surgical phases, Parkland levels, critical division actions, and CVS scores. This enables us to provide precise feedback on surgical information. Secondly, there must be a ‘visual’ platform that facilitates interaction between coaches and learners. However, there are currently no reports of such platforms worldwide. Therefore, our team has developed a SmartCoach platform. On this platform, not only can surgical reports be accessed, but coaches and coachees can also efficiently review and analyze surgeries using the platform’s video playback and key information timeline (Supplementary Fig. S2 in the Appendix 1, Supplemental Digital Content 1, http://links.lww.com/JS9/C817). This allows for effective postoperation review and analysis, thus achieving educational goals. In summary, the successful development and implementation of the two key technologies, ‘digitization’ and ‘visualization’ of surgeries, have paved the way for the successful implementation of this AI-assisted SmartCoach. Additionally, it has laid a solid foundation for the future expansion of many more surgical coaching projects.

Despite the growing interest and exploration of surgical coaching models by several research institutions, the lack of uniform standards is evident due to variations in research methods, coaching models, and outcome measures among different institutions^34,35^. Consequently, high-quality RCT evidence remains lacking regarding their specific clinical applications. On the one hand, most of the research on coaching models consists of nonrandomized interventional studies. Furthermore, these studies often use post-training questionnaires or interviews as the primary outcome measures to assess coachees’ satisfaction, self-confidence, self-assessment, and other subjective indicators after completing coaching training. This reliance on subjective measures not only raises concerns about the quality of evidence but also highlights the absence of objective assessments of skill improvement, limiting the direct applicability of these findings to clinical practice. On the other hand, surgical coaching research spans various professional disciplines, with most studies employing retrospective video analysis to review surgeries, while a minority adopts direct in-person coaching in the operating room. Although both approaches have shown improvements in surgical skills, they are highly dependent on expert resources and time. As a result, they are only conducted in a few centers and are challenging to scale up for widespread use^27,36^. In this prospective RCT, we innovatively incorporated AI throughout the coaching process. The application of intelligent surgical reports and review platforms not only eliminates the impact of subjective biases but also provides individualized and intelligent feedback. However, surgical coaching involves a complex workflow that includes multidimensional metrics. Although our AI algorithm can analyze surgical phases, disease severity, critical duct division action, and CVS score, a coach is still necessary to synthesize this data, devise a coaching plan, and communicate effectively with coachees. Thus, AI performed as an assistant to help coach and coachees. This significantly reduces the time required for organizing, reviewing, and analyzing surgical information. Consequently, coach and coachees can engage in comprehensive discussions regarding areas of technical improvement and goal enhancement within a limited time frame (30 min in this study). This ultimately leads to a significant improvement in safety and overall performance. It is worth noting that this study is a multicenter research project. Although participants were sourced from various medical institutions nationwide, all coaching activities were conducted online by one coach from one single institution, underscoring the scalability, and accessibility of this model. This demonstrates that AI-based coaching models not only greatly enhance the efficiency and effectiveness of surgical education but also significantly reduce the dependency on expert resources, making them highly promising for broader adoption.

### Limitations

Our study, while pioneering in its focus on AI-assisted surgical coaching, acknowledges several limitations. First, the limited sample size, due to the relatively small number of surgeons at the SmartHelp level, constrains the generalizability of our findings. Secondly, our coaching model still necessitates human coach involvement, which presents challenges for the routine implementation of coaching activities. Additionally, this study represents an initial exploration into the use of AI to assist in surgical coaching, given the technology’s current developmental stage. With the development of AI, fully automatic surgical education could be implemented in the future. Lastly, the scope of our study is restricted to a single type of surgery, raising questions about the applicability of our results to other surgical procedures. Future work will expand the focus to include multiple types of surgeries for a more comprehensive assessment.

## Conclusion

Our research provides compelling evidence that an AI-assisted LC coaching workflow can significantly enhance overall performance and safety among novice surgeons. All participants expressed a high level of satisfaction with the program, affirming its acceptability and practical feasibility. Based on these findings, we strongly encourage healthcare organizations to explore the implementation of AI-based coaching models across a diverse range of surgical procedures. While our results are promising, further studies, supported by high-level evidence, are essential to validate the efficacy and efficiency of AI-enabled surgical coaching.

## Ethical approval

The study was approved by the Ethics Committee on Biomedical Research, West China Hospital of Sichuan University (registration no. 2022-688).

## Source of funding

This work was supported by the Fund of the High-Quality Development of Guang’an People’s Hospital (21FZ001) and Regional Innovation Cooperation in Sichuan Province (2022YFQ0068).

## Author contribution

S.W., X.W., and M.T.: designed research, performed research, analyzed data, and wrote the paper; J.L., D.Q., Y.W., and S.Z.: developed the AI algorithm and revised the paper; E.B., Y.L., and C.W.: developed the AI algorithm and analyzed data; Y.X., G.L., F.G., and Y.C.: designed research and performed research; P.G., Z.W., H.C., and J.L.: analyzed data and performed research; Y.C., C.F., L.Y., and J.J.: refined AI algorithm and performed research; B.P.: designed research and revised the manuscript; H.W. and A.L.: designed research, performed research, analyzed data, and revised the paper. Manuscript was critically revised and approved by all authors.

## Conflicts of interest disclosure

The authors declare no conflicts of interest.

## Research registration unique identifying number (UIN)

http://www.chictr.org.cn/index.aspx. ChiCTR2300067363.

## Guarantor

Xin Wang.

## Supplementary Material

SUPPLEMENTARY MATERIAL
