# Supplementary material for: Impact of an AI-based laparoscopic cholecystectomy coaching program on the surgical performance: a randomized controlled trial
Source: Int J Surg. 2024 Jun 20;110(12):7816–23. doi: 10.1097/JS9.0000000000001798 (PMC11634122; doi:10.1097/JS9.0000000000001798)
Supplement: SUPPLEMENTARY MATERIAL [file js9-110-7816-s001.docx]

**Appendix 1**

Table S1 Changes from Baseline in Surgical Performance Between Coaching and Self-learning Participants

| Variables | LC-1 | | LC-2 | | LC-3 | | LC-4 | | LC-5 | |
| --- | --- | --- | --- | --- | --- | --- | --- | --- | --- | --- |
|  | Intervention | Control | Intervention | Control | Intervention | Control | Intervention | Control | Intervention | Control |
| LCRF | 31(29-32) | 34(31-37) | 31(30-33) | 33(31-34) | 34(32-37) | 31(31-38) | 39(37-40) | 34(32-38) | 40(39-41) | 38(32-39) |
| CVS score |  |  |  |  |  |  |  |  |  |  |
| <5 | 8(89%) | 9(100%) | 8(89%) | 8(89%) | 5(56%) | 8(89%) | 5(56%) | 8(89%) | 2(22%) | 8(89%) |
| ≥5 | 1(11%) | 0(0%) | 1(11%) | 1(11%) | 4(44%) | 1(11%) | 4(44%) | 1(11%) | 7(78%) | 1(11%) |
| Surgical Phase length |  |  |  |  |  |  |  |  |  |  |
| IT | 411(355-591) | 400(178-781) | 345(33-1181) | 444(112-882) | 548(282-2261) | 426(257-807) | 995(148-3277) | 530（66-2624） | 700(492-787) | 496(150-876) |
| EA | 85(63-122) | 101(66-108) | 98(52-158) | 76（28-115） | 73(0-123) | 108(0-152) | 70(16-258) | 82.5(0-112) | 82(63-129) | 100(80-108） |
| AL | 0（0-235） | 26(0-263) | 100(0-466) | 0(0-481） | 60(0-212) | 0(0-64) | 0(0-31) | 0（0-50） | 23(0-54) | 0(0-168） |
| MHT | 745(647-1037) | 990(753-1123) | 1245(469-1976) | 705(373-1349) | 582(277-1281) | 632(433-1308) | 702(269-2373) | 667(295-2759) | 546(438-656) | 492(430-684） |
| DGB | 337(215-401) | 163(151-300) | 232(0-998) | 176.5(83-418) | 115（0-718） | 138(88-285) | 201（0-704） | 182（80-498） | 188（66-474） | 134(101-322) |
| EG | 54（30-98） | 0（0-58） | 80（0-256） | 60（0-267） | 109（0-651） | 38（0-577） | 127（0-398） | 80.5（0-511） | 100（78-140） | 86(0-243） |
| COR | 253（152-532） | 362（173-538） | 215（0-1086） | 228(101-772) | 141(41-502) | 344(152-714) | 215(74-587) | 279（0-951） | 287（60-376） | 275(142-362） |
| Times of surgical phase switch | 19（14-20） | 15（10-21） | 15（5-27） | 17（8-28） | 14（7-26） | 12.5（9-19） | 13（8-24） | 13（5-32） | 10（10-16） | 10(10-14） |
| Effective operative time | 1998(1491-2510） | 1587(1390-2260) | 1960(687-3497) | 1642(858-2686) | 1318(428-2632) | 1404(722-1983) | 1487(498-3355) | 1344(681-4644) | 1251(890-1902) | 1320(891-1761) |
| iAE |  |  |  |  |  |  |  |  |  |  |
| Total | 16 | 8 | 16 | 4 | 5 | 10 | 8 | 21 | 15 | 16 |
| Bleeding | 15 (94) | 8 (100) | 13 (81) | 3 (75) | 4 (80) | 7 (70) | 8 (100) | 17 (81) | 13 (87) | 13 (81) |
| Injury | 1 (6) | 0 | 3 (19) | 1 (25) | 1 (20) | 3 (30) | 0 | 4 (19) | 2 (13) | 3 (19) |
| Cautery | 0 | 0 | 0 | 0 | 0 | 0 | 0 | 0 | 0 | 0 |
| Arrhythmia | 0 | 0 | 0 | 0 | 0 | 0 | 0 | 0 | 0 | 0 |

LCRF, laparoscopic cholecystectomy rating form; CVS, critical view of safety; IT, idle time; EA, establishing access; AL, adhesion lysis; MHT, mobilizing the hepatocystic triangle; DGB, dissecting gallbladder from liver bed; EG, extracting the gallbladder; COR, clear the operative region; iAE, intraoperative adverse event.

Table S2. Questionnaire Results

| Number of questions | Pre | | P* | Post | | P* | P^†^ | P^‡^ |
| --- | --- | --- | --- | --- | --- | --- | --- | --- |
|  | Intervention | Control |  | Intervention | Control |  |  |  |
| Q1 | 2.78 | 3.25 | 0.541 | 4.22 | 4.13 | 0.815 | **0.028** | 0.161 |
| Q2 | 2.56 | 3.25 | 0.370 | 4.78 | 4.13 | 0.114 | **0.011** | 0.263 |
| Q3 | 2.44 | 2.63 | 0.743 | 3.33 | 3.50 | 0.075 | 0.123 | 0.129 |
| Q4 | 1.89 | 2.00 | 0.963 | 3.89 | 3.50 | 0.370 | **0.007** | **0.033** |
| Q5 | 4.78 | 4.88 | 0.743 | 4.56 | 4.13 | 0.423 | 0.157 | 0.098 |
| Q6.1 | 4.33 | 3.75 | 0.370 | 4.33 | 3.88 | 0.277 | 1.000 | 0.655 |
| Q6.2 | 4.11 | 4.25 | 0.541 | 4.11 | 4.00 | 0.815 | 1.000 | 0.581 |
| Q6.3 | 4.11 | 4.00 | 0.963 | 3.78 | 4.13 | 0.423 | 0.429 | 0.564 |
| Q7 | 4.00 | 4.13 | 0.888 | 4.33 | 3.75 | 0.167 | 0.521 | 0.257 |
| Q8 | 4.11 | 3.88 | 0.606 | 4.22 | 3.88 | 0.370 | 0.891 | 1.000 |
| Q9 | 4.33 | 4.00 | 0.481 | 4.44 | 3.75 | 0.139 | 1.000 | 0.414 |
| Q10 | 4.56 | 4.25 | 0.743 | 4.33 | 3.88 | 0.277 | 0.854 | 0.180 |
| Q11 | 1.56 | 2.38 | 0.277 | N/A | N/A | N/A | N/A | N/A |
| Q12 | N/A | N/A | N/A | 4.44 | 4.00 | 0.370 | N/A | N/A |
| Q13 | N/A | N/A | N/A | 4.89 | 4.13 | 0.139 | N/A | N/A |

Q11 was only collected before the program; Q12 and Q13 were only collected after program. Questionnaire was demonstrated in the Appendix_2. *Comparison between two groups; ^†^Comparison of pre- and post-program questionnaire in coaching group; ‡Comparison of pre- and post-program questionnaire in self-learning group.

Table S3. Changes from Baseline in CVS Between Coaching and Self-learning Participants

| CVS score | LC-1 | | LC-2 | | LC-3 | | LC-4 | | LC-5 | | P^*^ | |
| --- | --- | --- | --- | --- | --- | --- | --- | --- | --- | --- | --- | --- |
|  | Intervention | Control | Intervention | Control | Intervention | Control | Intervention | Control | Intervention | Control | Intervention | Control |
| C1 |  |  |  |  |  |  |  |  |  |  |  |  |
| 0/2 | 0(0%) | 4(50%) | 0(0%) | 1(13%) | 0(0%) | 2(25%) | 0(0%) | 1(13%) | 0(0%) | 2(25%) | 0.157 | 0.340 |
| 1/2 | 6(67%) | 3(38%) | 8(89%) | 6(75%) | 4(44%) | 5(63%) | 7(78%) | 6(75%) | 2(22%) | 3(38%) |  |  |
| 2/2 | 3(33%) | 1(13%) | 1(11%) | 1(13%) | 5(56%) | 1(13%) | 2(22%) | 1(13%) | 7(78%) | 3(38%) |  |  |
| C2 |  |  |  |  |  |  |  |  |  |  |  |  |
| 0/2 | 7(78%) | 8(100%) | 6(67%) | 7(88%) | 4(44%) | 5(63%) | 4(44%) | 7(88%) | 2(22%) | 7(88%) | **0.025** | 0.317 |
| 2/2 | 2(22%) | 0(0%) | 3(33%) | 1(13%) | 5(56%) | 3(38%) | 5(56%) | 1(13%) | 7(78%) | 1(13%) |  |  |
| C3 |  |  |  |  |  |  |  |  |  |  |  |  |
| 0/2 | 1(11%) | 4(50%) | 0(0%) | 3(38%) | 0(0%) | 1(13%) | 0(0%) | 1(13%) | 0(0%) | 2(25%) | 0.257 | 0.260 |
| 1/2 | 5(56%) | 3(38%) | 5(56%) | 0(0%) | 6(67%) | 5(63%) | 4(44%) | 2(25%) | 4(44%) | 2(25%) |  |  |
| 2/2 | 3(33%) | 1(13%) | 4(44%) | 5(63%) | 3(33%) | 2(25%) | 5(56%) | 5(63%) | 5(56%) | 4(50%) |  |  |
| Total |  |  |  |  |  |  |  |  |  |  |  |  |
| <5 | 8(89%) | 9(100%) | 8(89%) | 8(89%) | 5(56%) | 8(89%) | 5(56%) | 8(89%) | 2(22%) | 8(89%) |  |  |
| ≥5 | 1(11%) | 0(0%) | 1(11%) | 1(11%) | 4(44%) | 1(11%) | 4(44%) | 1(11%) | 7(78%) | 1(11%) |  |  |

*Compassion of LC1 and LC5 in each group; C1=CVS I, C2=CVS II, C3=CVS III。


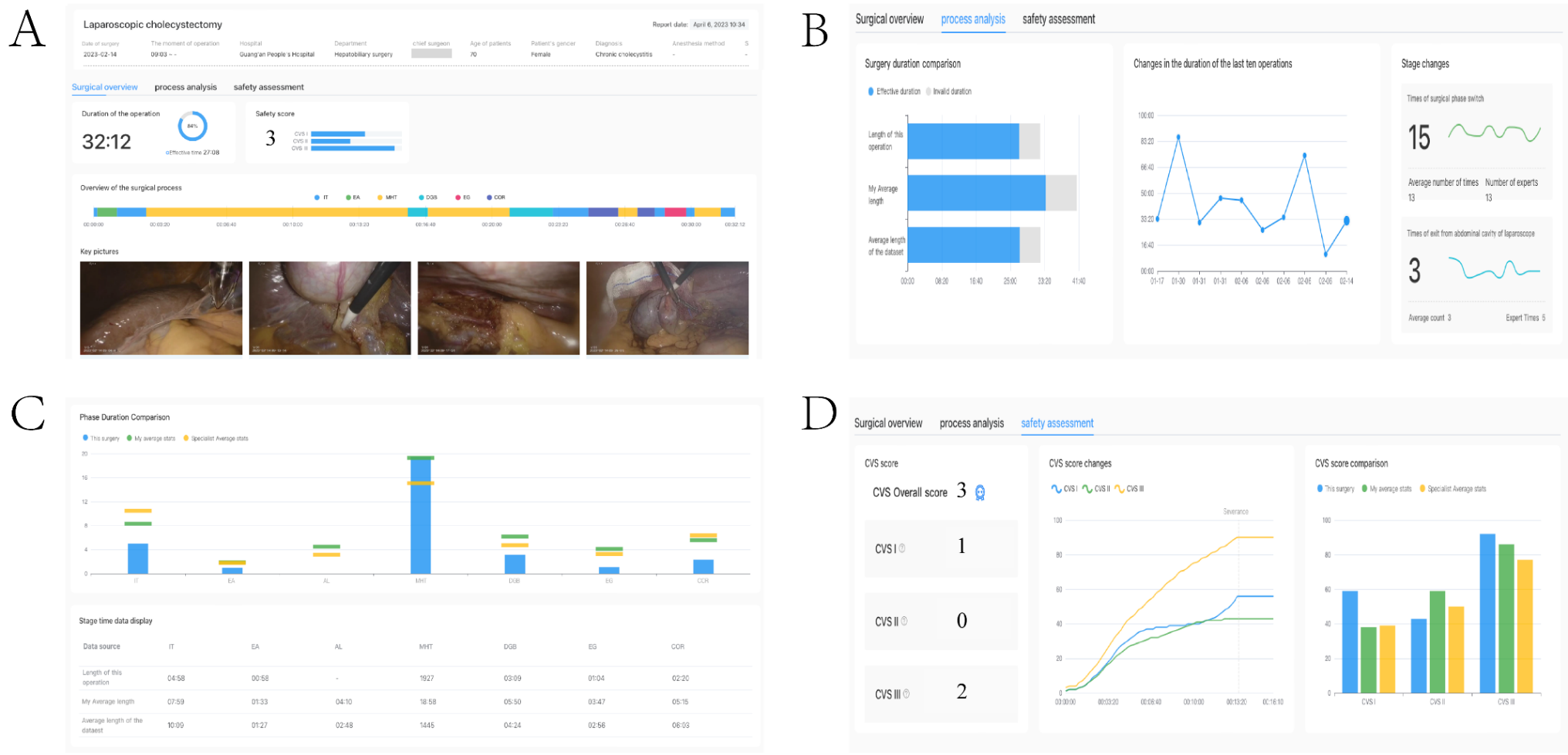


Figure S1. Example of SmartCoach surgical report. (A) Overview of surgical report. Duration of surgical phase was demonstrated. Safety score was calculated as CVS score. CVS I, CVS II, and CVS III were recorded as both total score of three criteria and percentage bar graph for each criteria (0-100, 100 was full mark). (B) Process analysis showed that the comparison of surgery duration, times of surgical phase switch, and times of exit from abdominal cavity for laparoscope. (C) Process analysis showed that the comparison of numerical surgical phase length in the current operation, surgeon’s average data, and average length of dataset. (D) Safety analysis showed CVS score for each criteria and total score, and CVS status changes during surgical process.


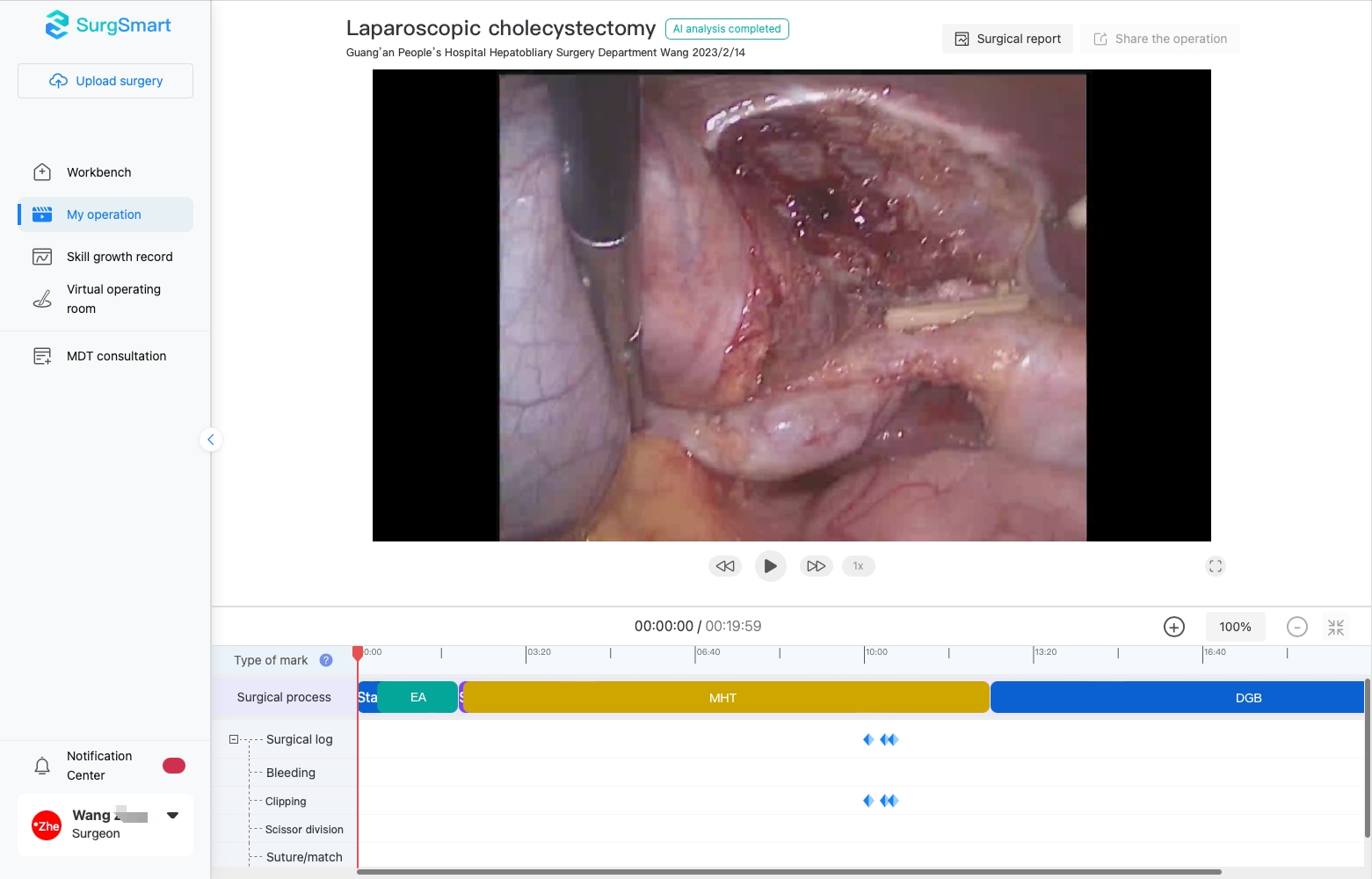


Figure S2. Online coaching interface.


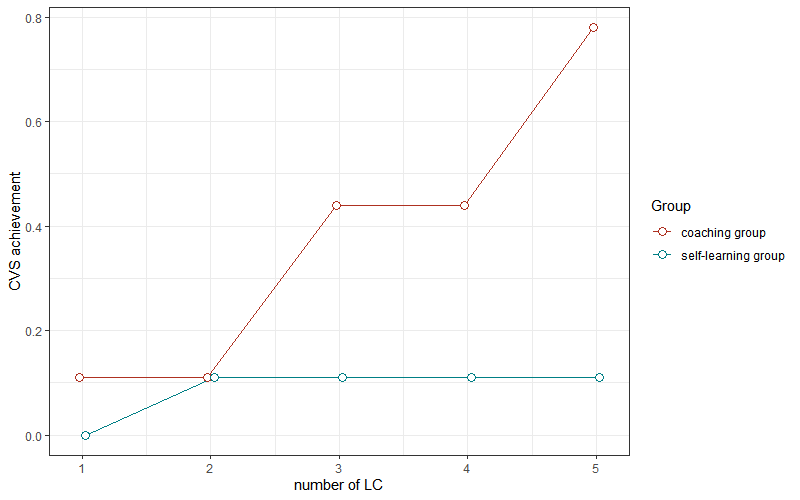


Figure S3. Changes of CVS achievement from baseline to the last LC for both arms. Red line is coaching group; green line is self-learning group. CVS, critical view of safety.
