# Supplementary material for: Impact of an AI-based laparoscopic cholecystectomy coaching program on the surgical performance: a randomized controlled trial
Source: Int J Surg. 2024 Jun 20;110(12):7816–23. doi: 10.1097/JS9.0000000000001798 (PMC11634122; doi:10.1097/JS9.0000000000001798)
Supplement: SUPPLEMENTARY MATERIAL [file js9-110-7816-s002.docx]

**Survey on Surgeon's Feedback for SmartCoach**

Dear Doctor,

We have designed the following survey to gather your feedback on the surgical coaching system. This questionnaire will be administered before and after participation to evaluate your perspective. Your input is crucial for enhancing the system's effectiveness.

**Basic Information**

1. Name: __________________________
2. Gender: _________________________
3. Title: ___________________________
4. Initial LC Surgery Volume: _________

**Pre- and Post Participation Section**

Please answer questions 1 to 11 before the participation.

1. **Are you familiar with the Critical View of Safety (CVS) in laparoscopic cholecystectomy (LC) and its clinical significance?**
2. Never heard of it
3. Rarely aware
4. Somewhat aware
5. Familiar
6. Clear understanding of each criterion
7. **Do you intentionally achieve CVS during your LC surgeries?**
8. Never
9. Rarely
10. Sometimes
11. Often
12. Always
13. **Are you familiar with AI applications in surgery?**
14. Never
15. Rarely
16. Sometimes
17. Often
18. Very familiar
19. **Have you heard of surgical coaching systems?**
20. Never
21. Rarely
22. Sometimes
23. Often
24. Very familiar
25. **Do you believe coaching systems like SmartCoach are needed for surgeons?**
26. Not needed
27. Slightly needed
28. Moderately needed
29. Highly needed
30. Extremely needed
31. **Please compare the surgical coaching system to other learning methods:**
32. **Compared to live lectures for basic knowledge:**
33. Significantly worse
34. Worse
35. Similar
36. Better
37. Much better

**(2) Compared to self-learning from surgery videos:**

1. Significantly worse
2. Worse
3. Similar
4. Better
5. Much better

**(3) Compared to participation in training courses (e.g., laparoscopic skill training):**

1. Significantly worse
2. Worse
3. Similar
4. Better
5. Much better
6. **Do you think the coaching system can be widely accepted by the medical community?**
7. Unlikely
8. Slightly unlikely
9. Moderately likely
10. Highly likely
11. Extremely likely
12. **Do you believe the coaching system can be extensively implemented?**
13. Unlikely
14. Slightly unlikely
15. Moderately likely
16. Highly likely
17. Extremely likely
18. **How much impact do you think this coaching system will have on future surgical practice?**
19. No impact
20. Slight impact
21. Moderate impact
22. Considerable impact
23. Significant impact
24. **Do you currently feel the need for surgical coaching?**
25. Not needed
26. Slightly needed
27. Moderately needed
28. Highly needed
29. Extremely needed

**Pre- Participation Section**

1. **Have you participated in any systematic surgical training programs?**
2. Never
3. Rarely
4. Sometimes
5. Often
6. Frequently

**Post-Participation Section**

Please answer questions 12 to 15 as you did before the participation.

1. **How do you comment on the AI-based surgical coaching system, SmartCoach?**
2. Insignificant
3. Slightly helpful, but limited
4. Moderately meaningful
5. Highly meaningful
6. Extremely meaningful
7. **How satisfied are you with this program?**
8. Very dissatisfied
9. Dissatisfied
10. Moderately Satisfied
11. Highly Satisfied
12. Extremely satisfied

Your participation and honest responses are greatly appreciated. Thank you for your time and input.

Sincerely,

[Your Name]

[Your Contact Information]
